# A Trajectory Close to the Anterolateral Vertebral Wall Becomes a Risk Factor for Intravascular Cement Leakage During Insertion of Cement-Augmented Fenestrated Pedicle Screws

**DOI:** 10.7759/cureus.88942

**Published:** 2025-07-28

**Authors:** Reimi Ikeda, Itsuo Shiina, Takane Nakagawa, Kazuhiro Ikeda, Hironori Takehashi, Takao Mizumachi, Ikuo Sugaya, Masao Koda, Hiroshi Takahashi

**Affiliations:** 1 Department of Orthopaedic Surgery, Moriya Daiichi General Hospital, Moriya, JPN; 2 Department of Orthopaedic Surgery, Institute of Medicine, University of Tsukuba, Tsukuba, JPN

**Keywords:** cement-augmented fenestrated pedicle screws, cement leakage, pulmonary cement embolism, risk factors, spinal fusion

## Abstract

Objective: We aim to identify the characteristics and risk factors of intravascular cement leakage (CL) in cement-augmented fenestrated pedicle screw (CAFPS) insertion, particularly focusing on screw trajectory, bone density, and vertebral body volume.

Methods: A retrospective observational study was conducted on 41 patients who underwent spinal fixation surgery with CAFPS from June 2022 to November 2024. Intravascular cement leakage (CL) was assessed using postoperative computed tomography (CT) scans. The relationship between CL occurrence and parameters including age, sex, insertion level, volume of bone cement injected, lumbar spine bone density, order of bone cement injection, vertebral volume, and screw to vertebral wall distance (SVD) was investigated. Univariate and multivariate analyses were performed to identify risk factors for intravascular CL.

Results: The incidence of intravascular CL was observed in 29 out of 64 vertebrae (45.3%) and 37 out of 128 CAFPS (28.9%). Multivariate analysis identified SVD as a significant independent risk factor for intravascular CL occurrence (p < 0.001, odds ratio: 0.71, 95% confidence interval (CI): 0.58-0.87). Receiver operating characteristic (ROC) curve analysis determined a cutoff value of 9.96 mm for SVD to predict intravascular CL occurrence.

Conclusion: A lower screw to vertebral wall distance was identified as a risk factor for intravascular CL. To reduce the incidence of intravascular CL, a steeper medial trajectory for CAFPS insertion might be beneficial as it could potentially increase the distance between the screw and the anterolateral wall of the vertebral body. This study included 41 patients and was retrospective in design. While the cutoff value of 9.96 mm for SVD may assist surgical planning, the findings should be interpreted with caution due to the limited sample size and lack of long-term clinical outcome data.

## Introduction

Pedicle screws (PS) are the most commonly used screws in posterior spinal fusion surgery. However, screw loosening is occasionally observed, especially in elderly patients with osteoporosis, resulting in poor surgical outcomes. Recent reports have shown that the incidence of PS loosening ranges from 1% to 15% in non-osteoporotic patients and can reach 10% to 60% in osteoporotic patients [1-4]. To enhance posterior fixation strength, various techniques such as sublaminar wiring, hook placement, and penetrating endplate screw have been utilized [5,6]. Recently, a novel technique called cement-augmented fenestrated pedicle screw (CAFPS) has been developed to enhance screw fixation strength in osteoporotic patients by injecting polymethylmethacrylate (PMMA) at the screw insertion site [7]. Biomechanical studies using cadaveric specimens have demonstrated that CAFPS have less toggling motion and higher pullout strength compared to conventional PS [8]. Clinical studies indicated significantly lower rates of screw loosening, reoperation, and postoperative pain scores with CAFPS compared to conventional PS [9]. However, cement leakage (CL) is a serious complication associated with PMMA usage, potentially leading to nerve injury, vessel damage, pulmonary cement embolism (PCE), and anaphylactic shock [7]. In balloon kyphoplasty (BKP), which is one of the other surgical procedures involving PMMA use, meta-analyses have shown that CL rates ranged from 18.4% to 54.7%, with several risk factors identified [10]. While some reports suggested that increasing the number of CAFPS insertions, higher cement injection volume, and spinal metastasis cases are associated with CL occurrence [11,12], there is a lack of detailed evaluation of the screw trajectory and vertebral body characteristics in relation to CL during CAFPS insertion. Therefore, we conducted a retrospective observational study to investigate the screw trajectory, bone density, and vertebral body volume in CAFPS insertion sites and identify the characteristics and risk factors of intravascular CL occurrence.

## Materials and methods

Patient population

**Figure 1:**
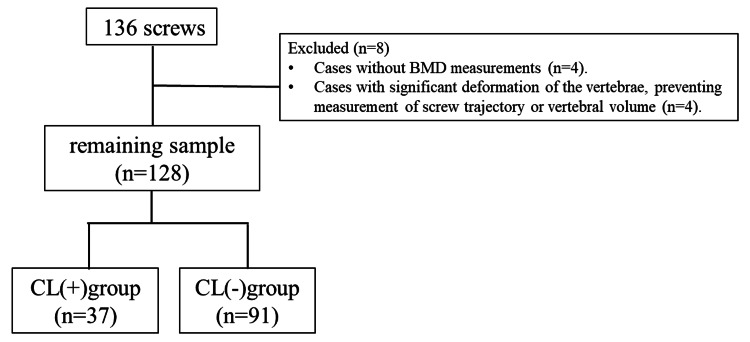
Exclusion criteria BMD: bone mineral density, CL: cement leakage

The present study was approved by the Institutional Review Board of Moriya Daiichi General Hospital (approval number: 2024-1, date: April 6, 2024). Informed consent for the participation and use of data was obtained from all patients. The study protocol conforms to the principles outlined in the 1964 Declaration of Helsinki. From June 2022 to November 2024, a total of 41 patients underwent spinal fixation surgery using CAFPS at our institution, involving 68 vertebral levels with 136 screws. CAFPS were utilized for vertebrae expected to have insufficient fixation strength with conventional pedicle screws due to severe osteoporosis. Cases in which bone density measurements were not conducted and vertebrae with significant deformities that prevented measurement of screw trajectory or vertebral volume (four vertebrae with eight screws) were excluded from the analysis. Ultimately, 40 cases involving 64 vertebral levels with 128 screws were analyzed in the present study (Figure 1).

Surgical procedure

All surgeries were performed under general anesthesia with patients in the prone position using two fluoroscopic units. The target conditions included 30 (75%) cases of acute vertebral fractures, one (2.5%) case of deformity healing following an old vertebral fracture, four (10%) cases of delayed healing of old vertebral fractures, one (2.5%) case of lumbar spondylolisthesis combined with acute vertebral fracture, one (2.5%) case of refracture at the same vertebral level following balloon kyphoplasty (BKP), two (5%) cases of adjacent vertebral fractures, and one (2.5%) case of adult spinal deformity. The surgical procedures included 33 (82.5%) cases of percutaneous vertebral stabilization (VBS) combined procedures, one (2.5%) case of posterior decompression, two (5%) cases of anterior lumbar interbody fusion combined procedures, two (5%) cases of percutaneous pedicle screw (PPS) removal and replacement (including screw exchange and extension of fixation range), one (2.5%) case combined with BKP, and one (2.5%) case of scoliosis surgery.

Thirty-nine (97.5%) cases underwent PPS placement, while one (2.5%) case underwent open screw insertion. In all cases, the screws were inserted via a transpedicular approach. The implants used were the Expedium Verse® or VIPER PRIME® Fenestrated Screw system from DePuy Synthes (Raynham, MA), and Vertecem＋ bone cement was used. The bone cement was allowed to sit for about 10 minutes after mixing to reach the appropriate viscosity before being carefully injected.

Assessment of cement leakage and measurement of imaging parameters

**Figure 2:**
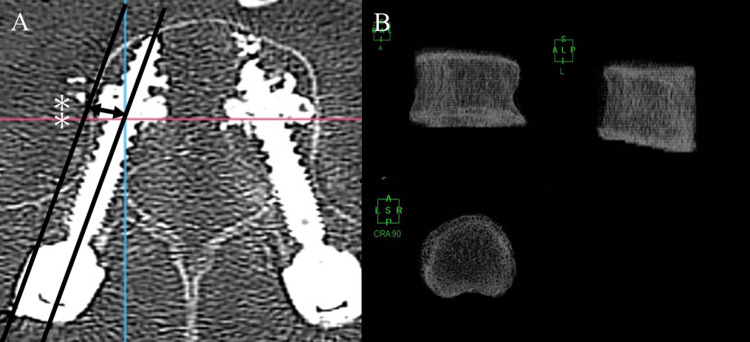
Measurement of imaging parameters (A) The asterisk represents the distance from the tangent line between the vertebral body wall and the screw insertion axis. (B) 3D vertebral body model from preoperative CT images. 3D: three-dimensional, CT: computed tomography

The presence of intravascular CL was assessed using horizontal computed tomography (CT) images taken one week postoperatively. Screw to vertebral wall distance (SVD) was measured to assess the CAFPS trajectory. SVD was defined as the distance from the tangent line between the vertebral side wall parallel to the screw insertion axis to the screw itself, reconstructed in cross-sectional images aligned with the screw insertion axis on postoperative CT scans (Figure 2A). CT reconstructions were performed using RadiAnt DICOM Viewer version 2023 (Medixant®, Poznan, Poland). To measure bone density, three-dimensional lumbar spine models of the screw-inserted vertebrae were created from preoperative CT images using the 3D analysis software AZE Virtual Place Raijin/Fujin (Canon Medical Systems Corporation®, Tochigi, Japan). Subsequently, vertebral volumes were extracted from the 3D images using Cutter Mode in the Volume editing tools (Figure 2B).

Analysis of risk factors for cement leakage

The group exhibiting intravascular CL was defined as the CL group, while the group without cement leakage was defined as the N group. Potential candidates for risk factors contributing to intravascular CL were investigated across both groups, including age, sex, insertion level, volume of bone cement injected, lumbar spine bone density, order of bone cement injection, vertebral volume, and SVD.

Statistical analysis

Results are presented as the mean ± standard deviation. A univariate analysis was conducted on the following parameters, divided into two groups based on the presence or absence of intravascular CL: age, lumbar spine bone density, vertebral volume, and SVD were analyzed using t-tests; the volume of bone cement was analyzed using the Mann-Whitney U test; and sex, insertion level, and order of cement injection were analyzed using Chi-square test. Multivariate analysis was performed on the variables that showed a trend in the univariate analysis. All statistical analyses were performed using the BellCurve for Excel version 4.06 software package (Microsoft Corp., Redmond, WA).

## Results

Patient characteristics and ratio of cement leakage

**Figure 3:**
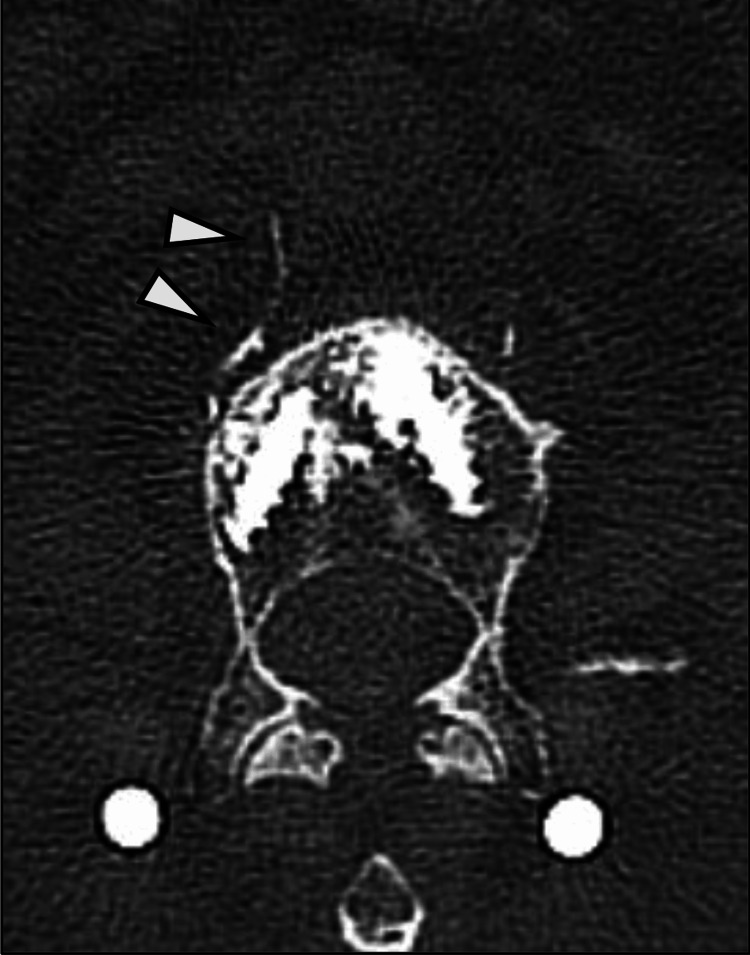
CT image showing cement leakage into the inferior vena cava CT: computed tomography

Patient characteristics are shown in Table 1. The volume of bone cement injected was 0.90 ± 0.13 mL. The incidence of intravascular CL was observed in 29 out of 64 (45.3%) vertebrae and 37 out of 128 (28.9%) screws. All cases of leakage were intravascular, with two cases noted to reach the inferior vena cava (IVC) (Figure 3), but symptomatic PCE was not observed.

**Table 1:** Patient characteristics VBS: vertebral body stent, PSF: posterior spinal fusion, OLIF: oblique lateral interbody fusion, PS: pedicle screw, BKP: balloon kyphoplasty, PCE: pulmonary cement embolism

Parameter	Value
Age	80.41 ± 8.37 (61-94)
Sex (male/female)	11/29
Implant	-
Expedium Verse Fenestrated Screw system®	10
VIPER PRIME Fenestrated Screw system®	30
Procedure	-
VBS + PSF	33
Decompression and fixation	1
Multilevel OLIF + PSF	2
PS removal and replacement	2
BKP + PSF	1
Scoliosis surgery	1
Amount of bone cement (mL)	0.90 ± 0.13 (0.5-1.0)
Incidence of intravascular CL	-
Vertebrae	29/64 (45.3%)
Screws	37/128 (28.9%)
Symptomatic PCE	None

Univariate analysis of factors associated with cement leakage

Univariate analysis of factors associated with cement leakage is shown in Table 2.

**Table 2:** Univariate analysis of factors associated with cement leakage CL: cement leakage, BMD: bone mineral density, SVD: screw to vertebral wall distance

Parameter	CL(+) (n=37)	CL(-) (n=91)	Chi-square (df)	P-value	Effect size (Cramer's V)
Age	79.11 ± 7.39	80.93 ± 8.72	-	0.265	-
Sex	-	-	2.182 (1)	0.140	0.131
Male	7	29	-	-	-
Female	30	62	-	-	-
Insertion level (thoracic or lumbar)	-	-	0.005 (1)	0.94	0.006
Thoracic	10	24	-	-	-
Lumbar	27	67	-	-	-
Amount of bone cement (mL)	0.90 ± 0.14	0.90 ± 0.12	-	0.78	-
Order of bone cement injection	-	-	0.469 (3)	0.926	0.061
No. 1	11	29	-	-	-
No. 2	12	28	-	-	-
No. 3	8	16	-	-	-
No. 4	6	18	-	-	-
BMD (g/cm^2^)	0.98 ± 0.23	0.91 ± 0.25	-	0.101	-
Vertebral body volume (cm³)	44.75 ± 11.59	49.33 ± 14.30	-	0.086	-
SVD (mm)	9.42 ± 2.55	11.80 ± 2.86	-	<0.001	-

SVD was significantly shorter in the CL group compared with the N group (p < 0.001). The vertebral body volume tended to be smaller in the CL-positive group, while lumbar bone mineral density tended to be higher in the same group.

There was a trend toward a higher proportion of first injections in the CL group, but the difference between groups was not statistically significant (p = 0.086 and p = 0.101).

Multivariate analysis of risk factors for cement leakage

**Figure 4:**
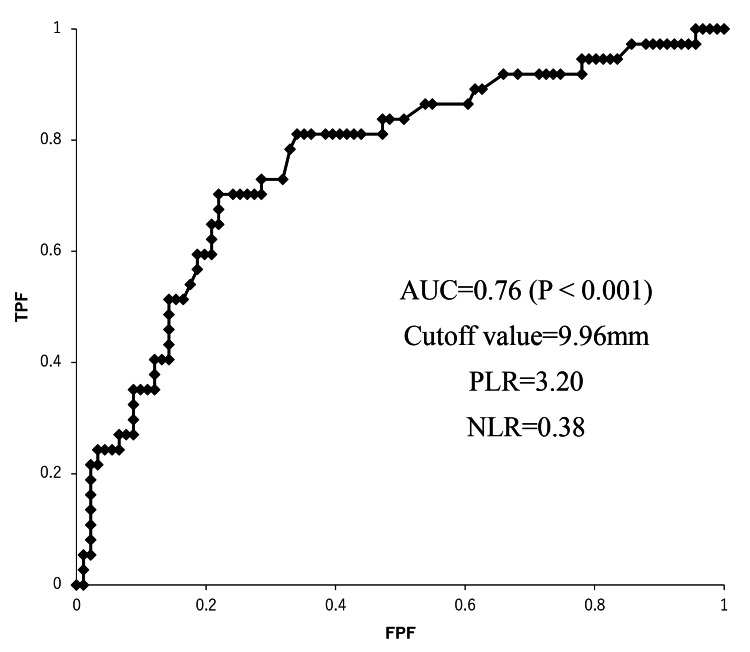
ROC curve of the relationship between SVD and the presence of intravascular CL SVD: screw to vertebral wall distance, CL: cement leakage, ROC: receiver operating characteristic, TPF: true positive fraction, FPF: false positive fraction, AUC: area under the curve, PLR: positive likelihood ratio, NLR: negative likelihood ratio

Based on the results of the univariate analysis, a multivariate analysis was conducted on lumbar spine bone density, vertebral body volume, and SVD (Table 3). As a result, SVD was identified as a significant independent risk factor (p < 0.001, odds ratio: 0.71, 95% confidence interval (CI): 0.58-0.87). Consequently, a receiver operating characteristic (ROC) curve was created to assess the relationship between SVD and the presence of intravascular CL (Figure 4). When the null hypothesis was set as an area of 0.5, the resulting p-value was <0.001, and the cutoff value for SVD was calculated to be 9.96 mm.

**Table 3:** Multivariate analysis of risk factors for cement leakage BMD: bone mineral density, SVD: screw to vertebral wall distance, CI: confidence interval

Variable	Odds ratio	95% CI	P-value
BMD (g/cm²)	1.3	0.21-7.42	0.799
Vertebral body volume (cm³)	1	0.97-1.04	0.876
SVD (mm)	0.71	0.58-0.87	<0.001

## Discussion

**Figure 5:**
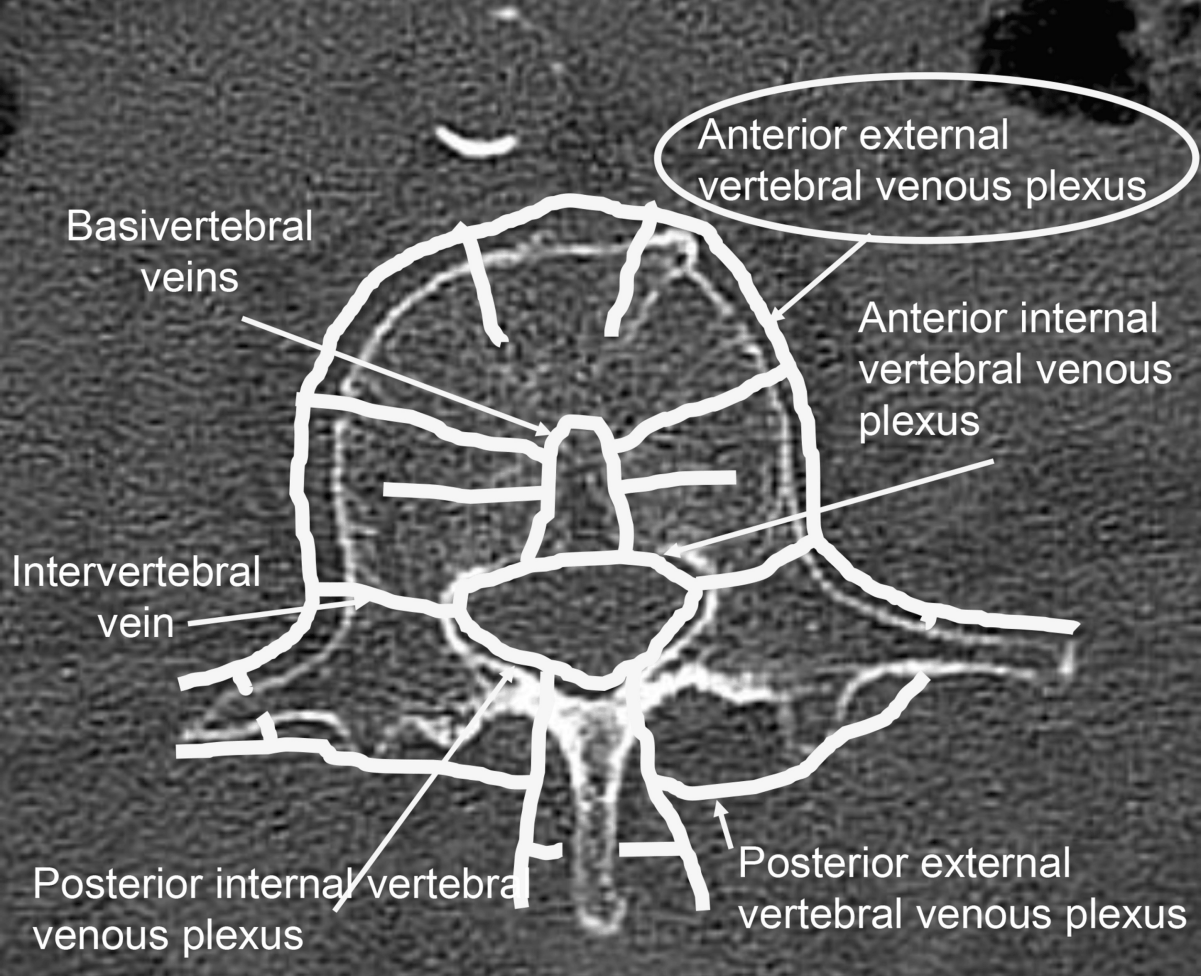
Schema of the vertebral venous plexuses

**Figure 6:**
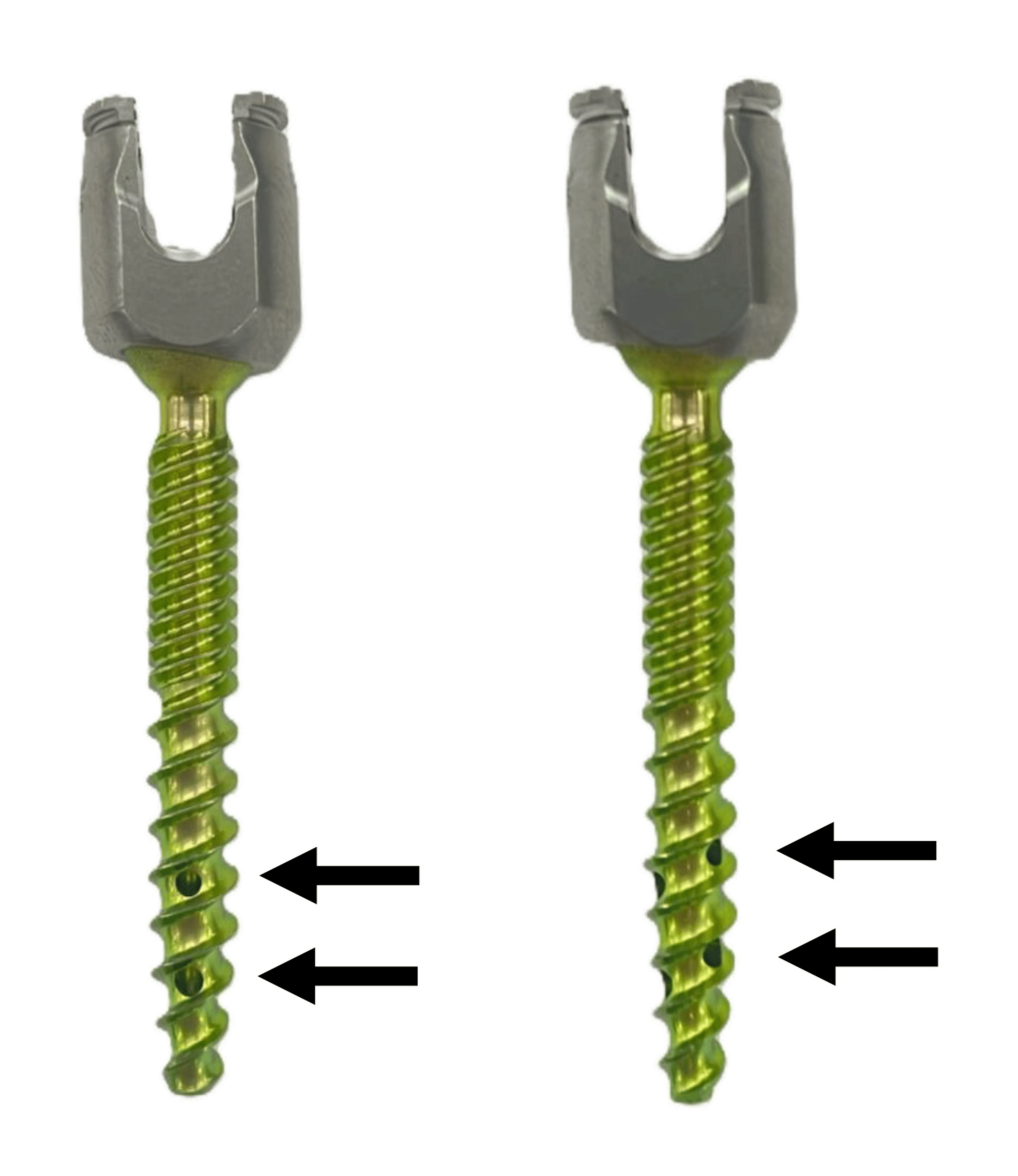
Screw design of CAFPS Arrows are cement holes positioned every 120°, with three holes located on the distal side of the screw and three on the proximal side. CAFPS: cement-augmented fenestrated pedicle screw

To our knowledge, the present study is the first to evaluate in detail the insertion trajectory of CAFPS and investigate its association with intravascular CL and to identify risk factors for intravascular CL. Recent reports indicated that the risk factors for CL in CAFPS include an increased number of CAFPS insertions, higher volumes of cement injection, and cases of spinal metastasis [11,12]. In addition, risk factors for PCE include high cement injection pressure, low cement viscosity, an increased number of CAFPS, larger volumes of cement injection, and thoracic level involvement [12-15]. Nevertheless, in the present study, SVD emerged as a significant risk factor for intravascular CL occurrence. As the mechanism by which intravascular CL is more likely to occur in CAFPS with lower SVD values, we hypothesize the following pathway of cement extravasation. A portion of the bone cement injected into the vertebral body enters the vertebral veins and then radiates into the anterior lateral vertebral venous plexus. From there, it may travel through the azygos venous system or directly enter the inferior vena cava. If cement ultimately reached the pulmonary arteries via this pathway, it can result in PCE (Figure 5) [16]. Actually, the fenestrated screws used in the present study had lateral holes at the distal end for cement extrusion (Figure 6). Therefore, as the distal end of the screw becomes close to the anterolateral aspect of the vertebral body that means lower SVD values, access to the anterolateral vertebral venous plexus becomes shorter, potentially leading to the occurrence of intravascular CL. In fact, in the present study, intravascular CL in 35 out of 37 (94.6%) CAFPS were confined to the anterolateral vertebral venous plexus, while in two cases, the cement reached the inferior vena cava (Figure 3). Regarding the incidence of CL, recent reports indicated that the pooled prevalence of CL in CAFPS is 34.4%, and the pooled risk is 38.3% [17]. In addition, the incidence of PCE has been reported to be 0%-5% [11,13,17]. The incidence in the present study was comparable to recent reports. Although no cases of PCE were observed in this study, it is crucial to recognize that PCE, despite often being asymptomatic, can result in serious complications, including right heart failure or sudden death [14]. To reduce the incidence of intravascular CL, a steeper medial trajectory for CAFPS insertion might be beneficial, as it could potentially increase the distance between the screw and the anterolateral wall of the vertebral body.

The present study has some limitations. First, this study only examined the risk of intravascular CL in the anterolateral region of the vertebral body and did not investigate the risk of CL into the spinal canal. Second, all screws used in this study were 45 mm long, so the effect of screw length variation on CL incidence could not be evaluated. It has been reported that cement injection into the posterior part of the vertebral body can lead to CL into the basivertebral and epidural veins [11], suggesting that using longer screws may be safer. Lastly, the sample size was small. Future studies should include a larger number of cases from multiple centers and conduct prospective observational research.

## Conclusions

We conducted a detailed evaluation of the screw trajectory in vertebrae with CAFPS insertion and investigated the characteristics and risk factors for intravascular CL occurrence. A lower SVD was identified as a risk factor for intravascular CL. To reduce the incidence of intravascular CL, we propose that CAFPS should be inserted with a steeper medial trajectory to maximize the distance between the screw and the anterolateral wall of the vertebral body.
